# Supplementary material for: Risk Perception and Fatigue in Port Workers: A Pilot Study
Source: Int J Environ Res Public Health. 2024 Mar 13;21(3):338. doi: 10.3390/ijerph21030338 (PMC10970156; doi:10.3390/ijerph21030338)
Supplement: Supplementary file 1 [file ijerph-21-00338-s001.zip › FigureS1.pdf]

Figure S1

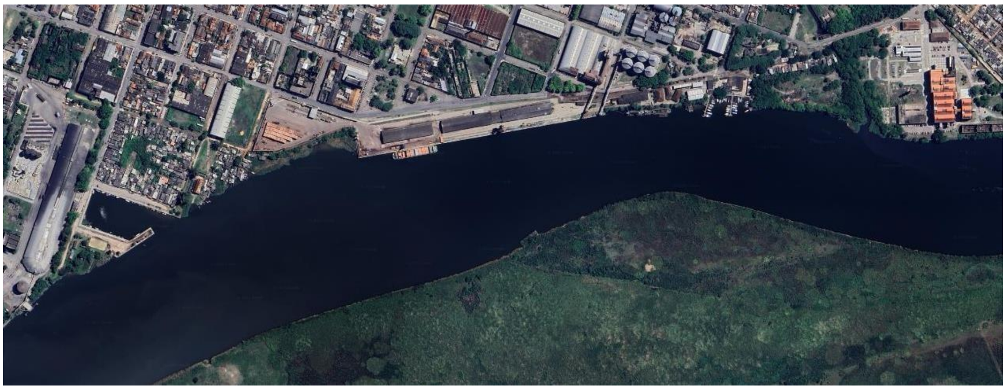

Google Earth version (2024). *Satellite image of the Port of Pelotas, Rio Grande do Sul, Brazil. São Gonçalo Channel – 31º46'55"S 52º20'00"W – 4m.* [Online] Available through: https://earth.google.com/web/search/pelotas/@-31.78207507,-52.33560491,4.18870804a,3030.58069998d,35y,0h,0t,0r/data=CigiJgokCdo4zbgT7DpAEdo4zbgT7DrAGafLa3uub0VAIT_sfImft1DAOgMKATA [Accessed on 15 November 2023].
