# Supplementary material for: Risk Perception and Fatigue in Port Workers: A Pilot Study
Source: Int J Environ Res Public Health. 2024 Mar 13;21(3):338. doi: 10.3390/ijerph21030338 (PMC10970156; doi:10.3390/ijerph21030338)
Supplement: Supplementary file 1 [file ijerph-21-00338-s001.zip › FigureS2.pdf]

Figure S2

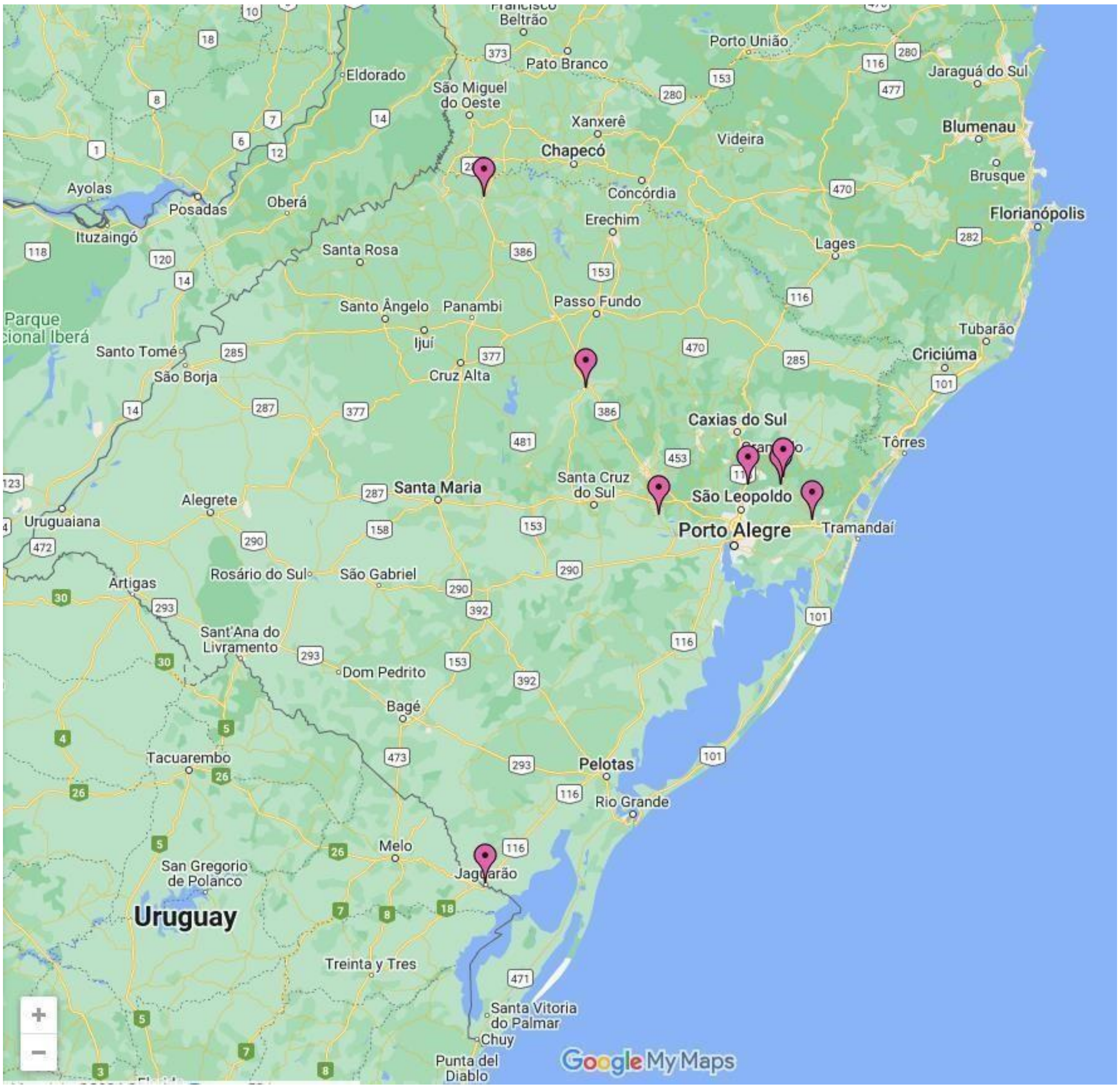

Google Maps (2024). Rio Grande do Sul, 1:100. Available from: https://www.google.com.br/maps/place/Rio+Grande+do+Sul/@-30.3853896,-56.3101946,7z/data=!3m1!4b1!4m6!3m5!1s0x9504720c40b45803:0xad9fb3dbaf9f73de!8m2!3d-29.3646459!4d-51.6657692!16zL20vMDFsXzlk?entry=ttu [Accessed on 15 November 2023].
