# Supplementary material for: Risk Perception and Fatigue in Port Workers: A Pilot Study
Source: Int J Environ Res Public Health. 2024 Mar 13;21(3):338. doi: 10.3390/ijerph21030338 (PMC10970156; doi:10.3390/ijerph21030338)
Supplement: Supplementary file 1 [file ijerph-21-00338-s001.zip › File 1.pdf]

File 1

Demographic and socioeconomic characteristics of Rio Grande do Sul (Source - Brazilian Institute of Geography and Statistics - https://cidades.ibge.gov.br/brasil/rs/panorama) and Pelotas (Source - Brazilian Institute of Geography and Statistics - https://cidades.ibge.gov.br/brasil/rs/pelotas/panorama).

Rio Grande do Sul

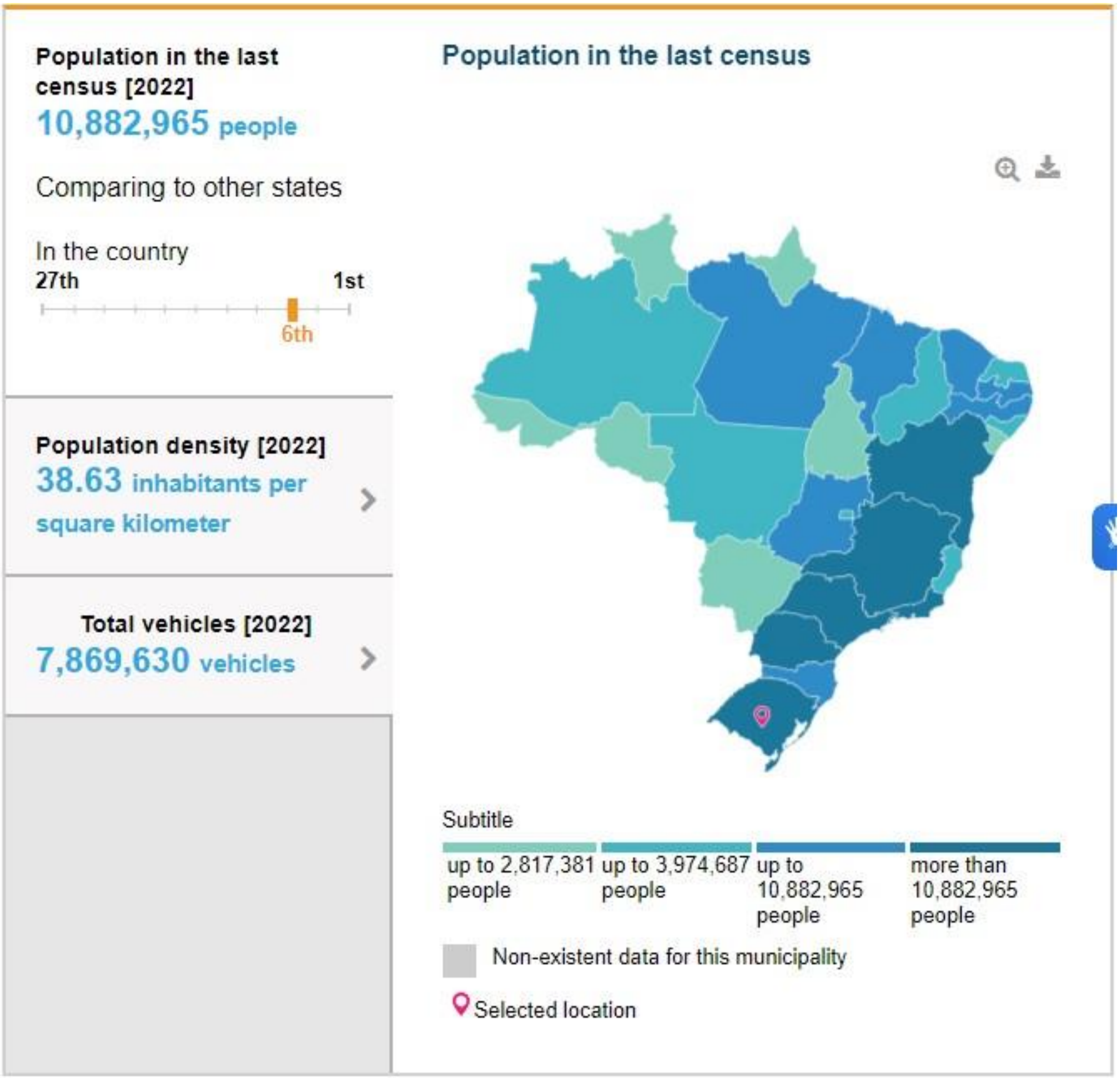

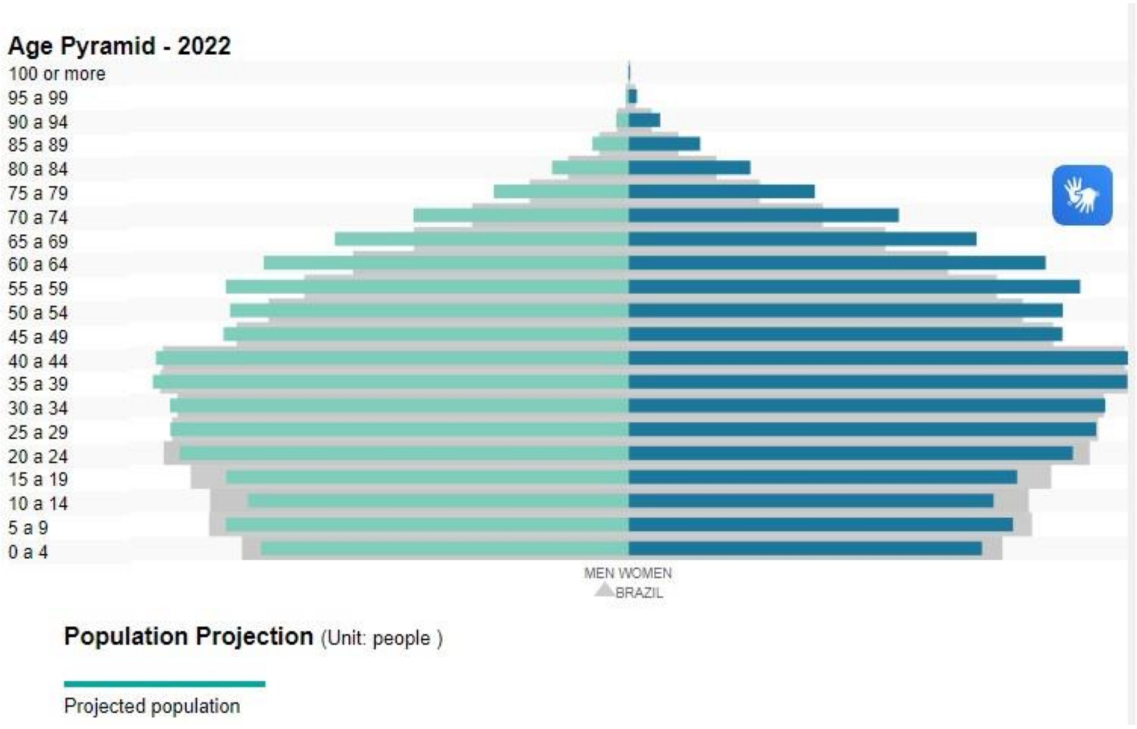

Age Pyramid - 2022
100 or more
95 a 99
90 a 94
85 a 89
80 a 84
75 a 79
70 a 74
65 a 69
60 a 64
55 a 59
50 a 54
45 a 49
40 a 44
35 a 39
30 a 34
25 a 29
20 a 24
15 a 19
10 a 14
5 a 9
0 a 4
MEN WOMEN
BRAZIL
Population Projection (Unit: people)
Projected population

Education

In 2021, the IDEB for the initial years of primary education in the public network was 5.8 and for the final years, 5. In comparison with other states, it was in positions 7 and 6 out of 27. The number of enrollments in education elementary school in 2021 had 1,257,992 enrollments, and secondary school enrollments had 346,363 enrollments. In comparison with other states, it was in positions 7 and 8 out of 27.

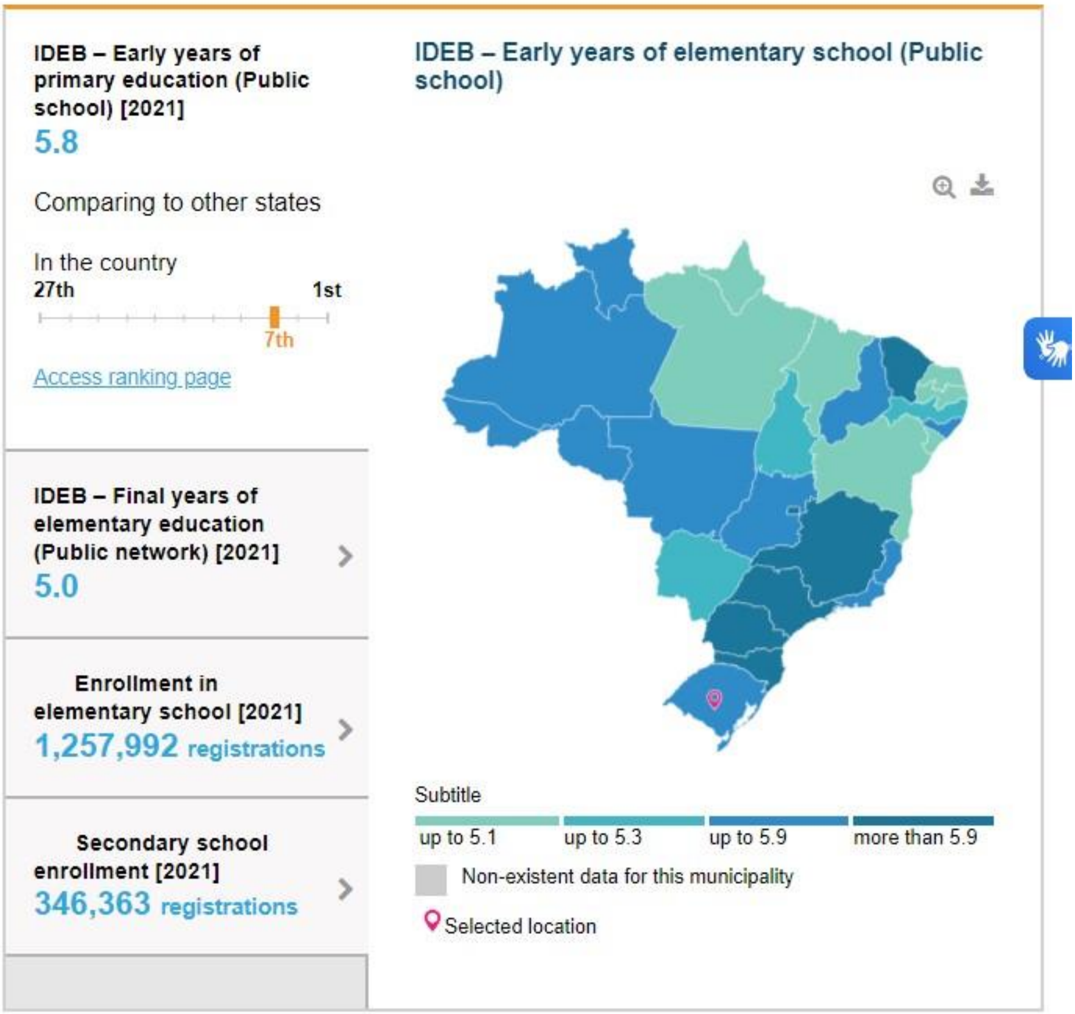

Work and Income

In 2022, the nominal monthly household income per capita was R$2,087, ranking 3rd among the 27 states. In 2016, the number of people aged 16 or over, employed in the reference week, was 5,842 people (x1000), ranking 5th among the 27 states. In 2016, the proportion of people aged 16 or over in formal work, considering only those employed in the reference week, was 67.6%, ranking 5th among the 27 states. In 2022, the proportion of people aged 14 or over employed in formal work in the reference week was 71.5%, ranking 2nd among the 27 states. In 2022, the average real usual income from the main job of people aged 14 or over, employed in formal work in the reference week was R$2,938, ranking 7th among the 27 states. In 2021, the number of people employed in public administration, defense and social security was 360,276 people, ranking 7th among the 27 states.

Nominal monthly household income per capita [2022]
2.087 R$

Comparing to other states

In the country
27th 1st
3rd

People aged 16 or over employed in the reference week [2016]
5,842 people (×1000)

Proportion of people aged 16 or over in formal work, considering only those employed in the reference week [2016]
67.6 %

Proportion of people aged 14 or over, employed in formal work in the reference week [2022]
71.5 %

Average usual real income from the main job of people aged 14 or over, employed in formal work in the reference week [2022]
2.938 R$

Personnel employed in public administration, defense and social security [2021]
360,276 people

Nominal monthly household income per capita

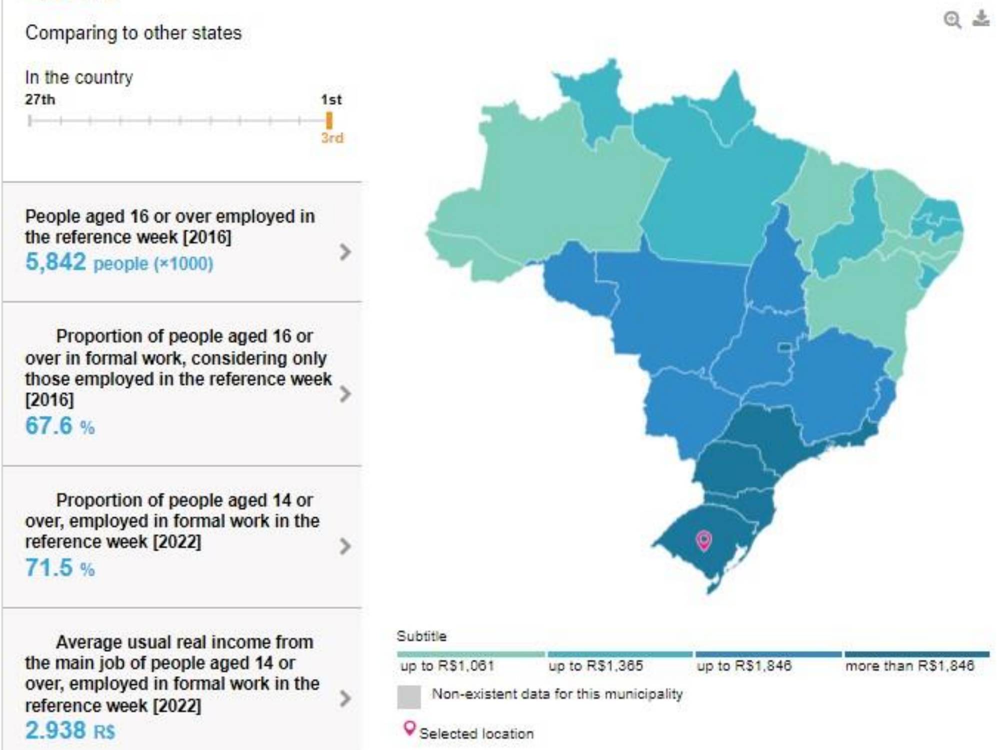

Economy

In 2021, the HDI was 0.771, ranking 5th among the 27 states. The value of budget revenues in 2017 was R$ 66,397,468.18 (x1000), and the value of committed budget expenses was R$ 62,476,279.34 (x1000), ranking 4 and 4 among the 27 States.

Human Development Index (HDI) [2021]
0.771

Comparing to other states

In the country
27th — 5th — 1st

Access ranking page

Budget revenues realized [2017]
66.397.468,18 R$ (×1000)

Committed budget expenditures [2017]
62.476.279,34 R$ (×1000)

Human Development Index (HDI)

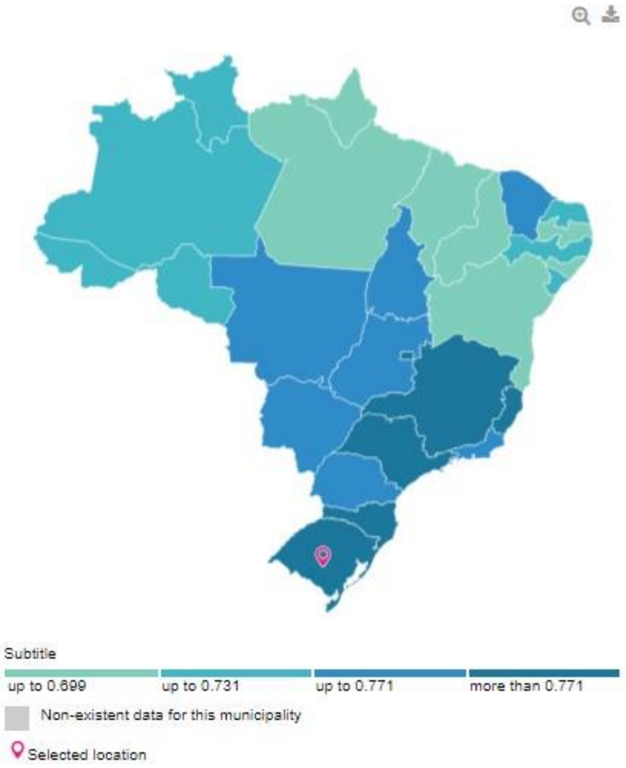

Territory

The state's territorial area in 2022 was 281,707.151 km², placing it in position 9 among the 27 states, and the number of municipalities was 497, placing it in position 3 among the 27 states. The urbanized area in 2019 was 3,601.63 km², which placed it in position 3 among the 27 states.

Number of municipalities [-]
497 municipalities

Comparing to other states

In the country
27th — 1st
3rd

Area of the territorial unit [2022]
281.707,151 km²

Urbanized area [2019]
3.601,63 km²

Number of municipalities

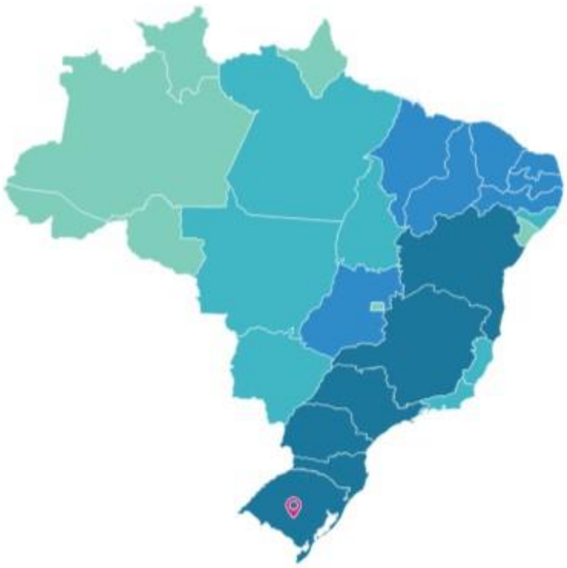

Subtitle

up to 78 municipalities | up to 167 municipalities | up to 295 municipalities | more than 295 municipalities

Non-existent data for this municipality

Selected location

Pelotas

Population

In 2022, the population was 325,685 inhabitants and the demographic density was 202.44 inhabitants per square kilometer. In comparison with other municipalities in the state, it was in positions 4 and 65 out of 497. In comparison with municipalities across the country, it was in positions 86 and 785 out of 5570.

Population in the last census [2022]
325,685 people

Comparing to other municipalities

In the country
5570º — 1st — 86th

In the State
497th — 1st — 4th

In the immediate geographic region
17th — 1st — 1st

Population density [2022]
202.44 inhabitants per square kilometer

Population in the last census

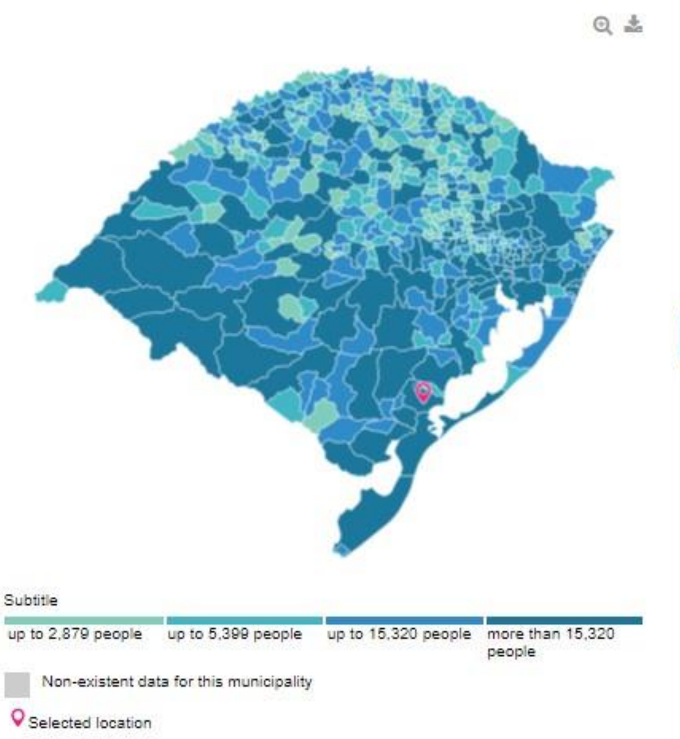

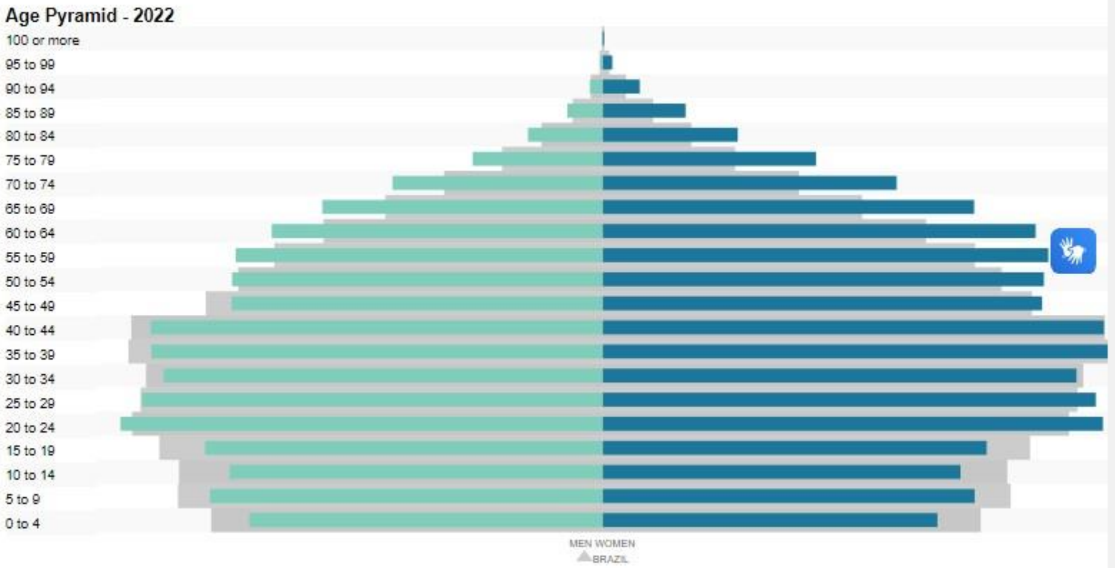

Work and Income

In 2021, the average monthly salary was 2.8 minimum wages. The proportion of employed people in relation to the total population was 23.98%. In comparison with other municipalities in the state, it occupied positions 41 out of 497 and 184 out of 497, respectively. In comparison with cities across the country, it ranked 248 out of 5570 and 1072 out of 5570, respectively. Considering households with monthly income of up to half a minimum wage per person, 31.9% of the population had these conditions, which placed it in position 229 out of 497 among the cities in the state and in position 4310 out of 5570 among cities in Brazil.

Average monthly salary of formal workers [2021]
2.8 minimum wages

Comparing to other municipalities

In the country
5570° — 1st
248th

In the State
497th — 1st
41st

In the immediate geographic region
17th — 1st
2nd

Access ranking page

Busy people [2021]
82,436 people

Employed population [2021]
23.98 %

Percentage of the population with nominal monthly per capita income of up to 1/2 minimum wage [2010]
31.9 %

Average monthly salary of formal workers

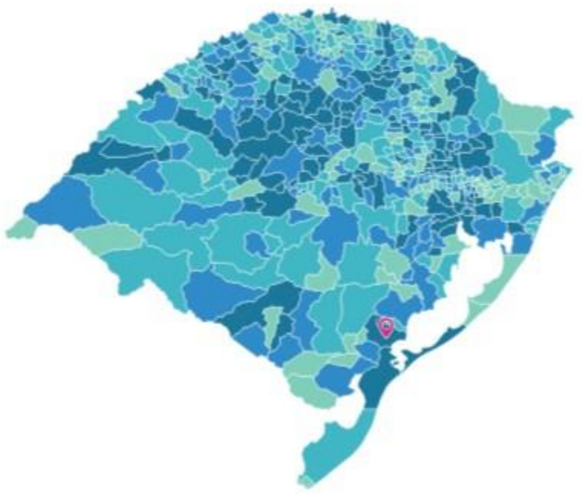

Subtitle

up to 2.1 minimum wages | up to 2.3 minimum wages | up to 2.5 minimum wages | more than 2.5 minimum wages

Non-existent data for this municipality

Selected location

Education

In 2010, the school enrollment rate for 6 to 14 year olds was 96.9%. In comparison with other municipalities in the state, it was in position 394 out of 497. In comparison with municipalities across the country, it was in position 3751 out of 5570. In relation to IDEB, in 2021, IDEB for the initial years of education fundamental in the public network was (no data) and for the final years, 5. In comparison with other municipalities in the state, it was in positions (no data) and 238 out of 497. In comparison with municipalities across the country, was in positions (no data) and 1937 of 5570.

Schooling rate from 6 to 14 years of age [2010]
96.9 %

Comparing to other municipalities

In the country
5570° — 1st
3751st

In the State
497th — 1st
394th

In the immediate geographic region
17th — 1st
13th

IDEB – Early years of primary education (Public school) [2021]
-

IDEB – Final years of elementary education (Public network) [2021]
5.0

Schooling rate from 6 to 14 years of age

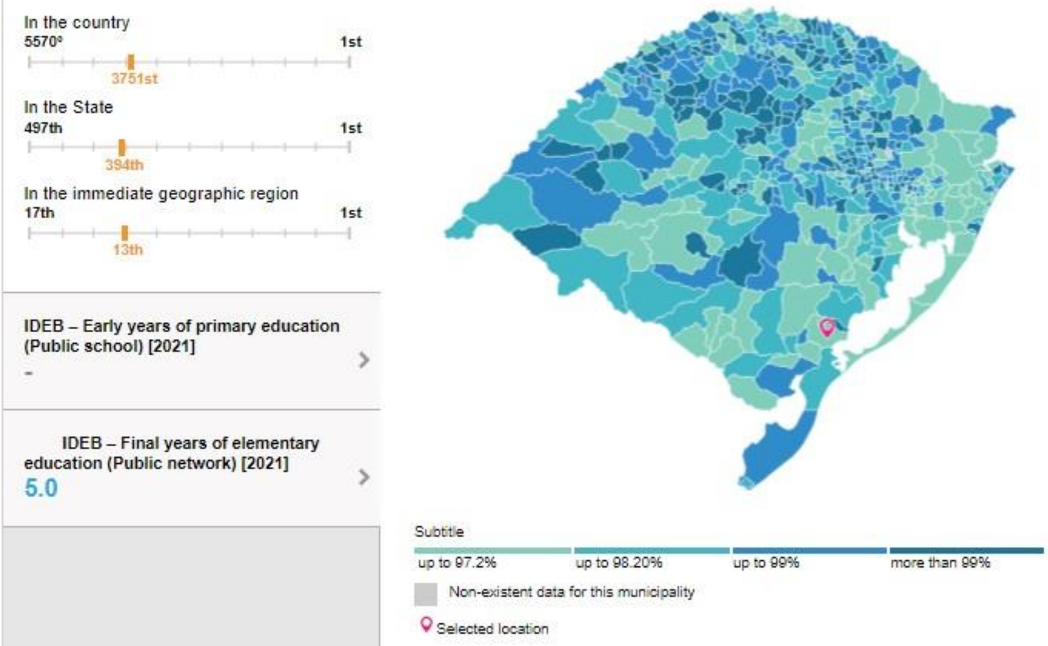

Economy

In 2021, GDP per capita was R$31,347.6. In comparison with other municipalities in the state, it ranked 413 out of 497 among the state's municipalities and 2026 out of 5570 among all municipalities. The percentage of external revenue in 2015 was 61.4%, which placed it in position 456 out of 497 among the state's municipalities and 4784 out of 5570. In 2017, the total revenue generated was R$ 971,193.15 (x1000) and the total committed expenses was R$906,719.1 (x1000). This leaves the municipality in positions 4 and 4 out of 497 among the state's municipalities and in 82 and 76 out of 5570 among all municipalities.

GDP per capita [2021]
31,347.60 R$

Comparing to other municipalities

In the country
5570° — 1st
2026th

In the State
497th — 1st
413th

In the immediate geographic region
17th — 1st
12th

Access ranking page

Percentage of revenue from external sources [2015]
61.4 %

Total revenue realized [2017]
971,193.15 R$ (×1000)

Total committed expenses [2017]
906,719.10 R$ (×1000)

GDP per capita

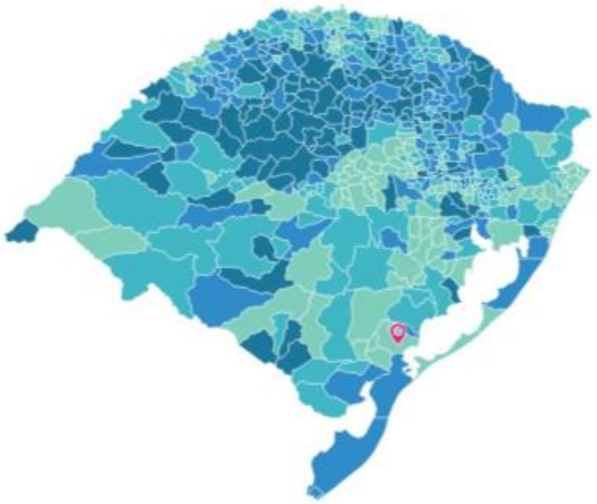

Subtitle

up to R$35,227.12 | up to R$47,482.38 | up to R$66,163.47 | more than R$66,163.47

Non-existent data for this municipality

Selected location

Health

The average infant mortality rate in the city is 9.07 per 1,000 live births. Hospitalizations due to diarrhea are 0.1 for every 1,000 inhabitants. Compared to all municipalities in the state, it is ranked 156 out of 497 and 395 out of 497, respectively. When compared to cities across Brazil, these positions are 3007 out of 5570 and 4734 out of 5570, respectively.

Infant Mortality [2020]

9.07 deaths per thousand live births

Comparing to other municipalities

In the country
5570º ... 3007th ... **1st**

In the State
497th ... 156th ... **1st**

In the immediate geographic region
17th ... 8th ... **1st**

Access ranking page

Hospitalizations for diarrhea [2016]

0.1 hospitalizations per thousand inhabitants

Child mortality

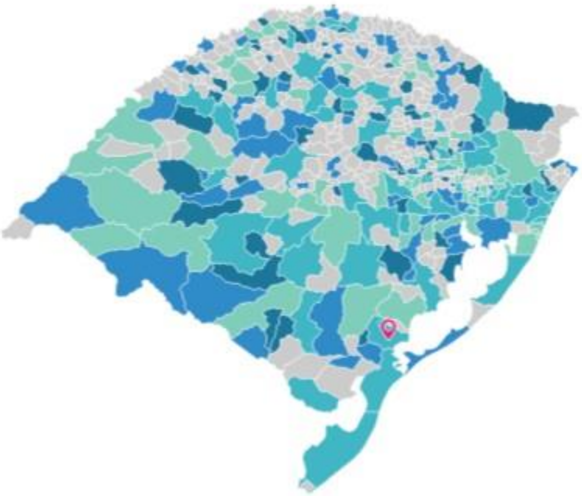

Subtitle

up to 7.81 deaths per thousand live births	up to 12.62 deaths per thousand live births	up to 21.28 deaths per thousand live births	more than 21.28 deaths per thousand live births

Non-existent data for this municipality

Selected location

Environment

It presents 82.4% of households with adequate sewage, 84.6% of urban households on public roads with trees and 34.4% of urban households on public roads with adequate urbanization (presence of drains, sidewalks, paving and curbs).). When compared to other municipalities in the state, it is ranked 71st out of 497, 243rd out of 497 and 120th out of 497, respectively. When compared to other cities in Brazil, its position is 839 out of 5570, 2020 out of 5570 and 1000 out of 5570, respectively.

Urbanized area [2019]
79.39 km²

Comparing to other municipalities

In the country
5570° … 1st
71st

In the State
497th … 1st
4th

In the immediate geographic region
17th … 1st
1st

Adequate sewage system [2010]
82.4 %

Afforestation of public roads [2010]
84.6 %

Urbanization of public roads [2010]
34.4 %

Population exposed to risk [2010]
53,470 people

Urbanized area

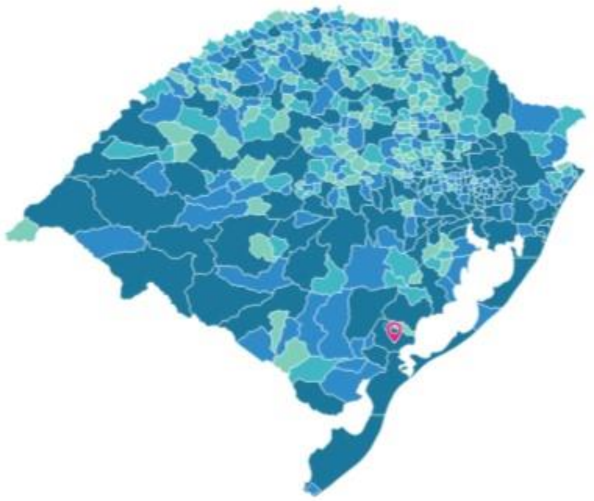

Subtitle

up to 1.21 km² | up to 2.5 km² | up to 7.3 km² | more than 7.3 km²

Non-existent data for this municipality

Selected location

Territory

In 2022, the municipality's area was 1,608.78 km², which places it in position 41 out of 497 among the state's municipalities and 920 out of 5570 among all municipalities.

Area of the territorial unit [2022]
1,608,780 km²

Comparing to other municipalities

In the country
5570º — 1st — 920º

In the State
497th — 1st — 41st

In the immediate geographic region
17th — 1st — 9th

Area of the territorial unit

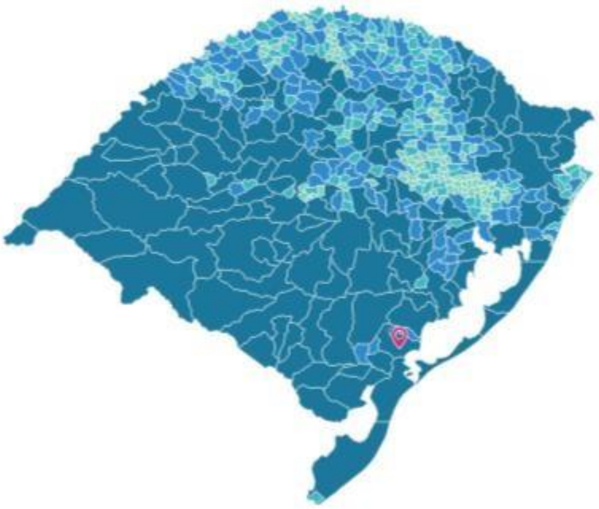

Subtitle

up to 125.62 km² | up to 238,082 km² | up to 505,983 km² | more than 505,983 km²

Non-existent data for this municipality

Selected location
